# Noncoding RNA therapeutics for substance use disorder

**DOI:** 10.3389/adar.2022.10807

**Published:** 2022-12-20

**Authors:** Seyed Afshin Seyednejad, Gregory C. Sartor

**Affiliations:** 1 Department of Pharmaceutical Sciences, University of Connecticut, Storrs, CT, United States,; 2 Connecticut Institute for the Brain and Cognitive Sciences (CT IBACS), Storrs, CT, United States

**Keywords:** microRNA, substance use disorder, noncoding RNA, lncRNA, SMIRNA, circRNA

## Abstract

Although noncoding RNAs (ncRNAs) have been shown to regulate maladaptive neuroadaptations that drive compulsive drug use, ncRNA-targeting therapeutics for substance use disorder (SUD) have yet to be clinically tested. Recent advances in RNA-based drugs have improved many therapeutic issues related to immune response, specificity, and delivery, leading to multiple successful clinical trials for other diseases. As the need for safe and effective treatments for SUD continues to grow, novel nucleic acid-based therapeutics represent an appealing approach to target ncRNA mechanisms in SUD. Here, we review ncRNA processes implicated in SUD, discuss recent therapeutic approaches for targeting ncRNAs, and highlight potential opportunities and challenges of ncRNA-targeting therapeutics for SUD.

## Introduction

Substance use disorder (SUD) continues to be a worldwide public health crisis (1). Although many of the underlying mechanisms that drive compulsive drug use have been elucidated, the number of pharmacological agents that are approved to treat SUD remains stagnant (2). Current pharmacotherapies for SUD largely consist of small molecule modulation of neurotransmitter receptor activity (2). While these treatments have shown some clinical success, many promising therapeutic opportunities will likely be missed if this narrow focus continues. Thus, to move the field forward and to improve patient outcomes, novel pharmacological interventions for SUD are greatly needed.

As only 1%–2% of the human genome encodes for protein (3, 4) and many proteins lack druggable sites for small molecules (5), researchers are turning to nucleic acid-based treatments to target previously undruggable mechanisms. The recent progress in nucleic acid chemistry, bioinformatic approaches, and delivery systems has dramatically improved several issues associated with stability, specificity, and tolerability of RNA-targeting drugs (6). These advancements have resulted in successful clinical trials and recent approvals of nucleic acid-based therapeutics by the Food and Drug Administration (FDA) and the European Medicines Agency (EMA) for various disorders (7, 8). Additional factors contributing to the rising interest and growth in nucleic acid-based therapeutics include rationale design, rapid optimization and adaptability to evolving targets, high selectivity, and potentially longer half-life leading to infrequent administration (7, 8). While many of these initial therapies aimed to modulate protein-coding transcripts, more recently, there has been a rising interest in developing nucleic acid-based drugs that target noncoding RNAs (ncRNAs), given their significant roles in cell type-specific biological processes in both health and disease (9).

In animal models of SUD, several ncRNAs have been shown to play functional roles in drug-seeking behaviors (10), and in humans, many genetic variants linked to SUD are located within noncoding regions of the genome (11). Thus, as the number of putative ncRNA targets in SUD continues to grow, nucleic acid-based therapeutics will likely be required to modulate these novel mechanisms. In this review, we describe different ncRNA classes involved in SUD, provide an overview of the modalities used to manipulate ncRNAs, and highlight ncRNA-based treatment strategies for SUD. We also discuss the ongoing challenges of ncRNA targeting and provide future perspectives for ncRNA-based therapeutics in SUD.

### Noncoding RNAs in SUD

In humans and other primates, ncRNA expansion has fostered the intricate regulatory network required for brain evolution and cognitive advancement (12). ncRNAs are abundantly expressed in the central nervous system (CNS) where many are transcribed in a cell type-specific manner (13). In neuropsychiatric disorders, including SUD, changes in brain ncRNA expression have been associated with disease pathophysiology (13, 14), and several ncRNAs have been functionally examined in CNS disease models (15–17). In SUD, most of the research has focused on 3 classes of ncRNAs: microRNAs (miRs), long noncoding RNAs (lncRNAs), and more recently circular RNAs (circRNAs) (Table 1). In this section, we briefly review the mechanistic roles of miRs, lncRNAs, and circRNAs, and highlight potential therapeutic ncRNA targets in SUD.

### MicroRNAs

MicroRNAs are a class of small noncoding RNAs with a highly conserved single-stranded sequence of approximately 22 nucleotides (36). Initially, miRs are transcribed into longer primary transcripts, called pri-miRs. The pri-miR is then cleaved by Drosha in the nucleus to produce the precursor miR (pre-miR) before being processed by Dicer in the cytosol to yield the mature miR. The mature miR is then loaded into the RNA-induced silencing complex (RISC) where it hybridizes to the three prime untranslated region (3′-UTR) of target mRNAs to mediate translational inhibition, cleavage, or degradation (36). With the ability to modulate 20%–50% of protein-coding genes, miRs are considered master regulators of many cellular activities (37–39). Notably, miRs play essential roles in brain development and neuroplasticity, and their dysregulation has been linked to the pathophysiology of most neuropsychiatric disorders (40–42).

In preclinical and clinical SUD studies, many miRs are dysregulated in reward-related brain regions following cocaine (25, 26, 43–48), amphetamine (49–51), methamphetamine (28–30, 52–57), nicotine (34, 58–63), opioid (31, 32, 64–71), and alcohol use (19, 20, 22, 72–83). SUD-associated miRs and their underlying mechanisms have been thoroughly reviewed elsewhere (14, 84). Of the miRs correlated with drug use, several have been shown to regulate the expression of known SUD targets that play important roles in maladaptive neuroplasticity and drug-seeking behaviors (e.g., *BDNF*, *CREB*, *MeCP2*, *CaMKIIa*) (14). In particular, miR-212, miR-132, miR-181, miR-9, and let-7 may be of interest for clinical targeting as altered expression of these miRs has been observed across multiple drugs of abuse in human and animal samples (14). In addition to miR activity in the brain, miR levels in SUD patient blood samples have been correlated with drug history and relapse (23, 85–94). Thus, circulating miRs may be a useful auxiliary measurement for diagnosis and treatment.

While there have been no clinical trials using miR-targeting therapeutics in SUD patients, several miRs have been explored functionally in preclinical SUD models (Table 1). For example, viral-mediated overexpression of miR-124a in the dorsolateral striatum enhanced alcohol-induced conditioned place preference (CPP) and increased alcohol intake, while silencing its expression attenuated CPP and alcohol consumption (20). In cocaine CPP experiments, overexpression of miR-124 and let-7d in the nucleus accumbens (NAc) attenuated cocaine CPP, whereas miR-181a overexpression enhanced CPP (95). The opposite effect on cocaine CPP was observed following knockdown of miR-124, let-7d, and miR-181a in the NAc. In self-administration studies, overexpression of miR-212 in the dorsal striatum attenuated compulsive cocaine intake in the extended-access self-administration procedure (25). Consistent with these observations, reduced levels of miR-212 in the striatum were associated with cocaine intake in addiction-prone but not addiction-resistant rats (96). In opioid self-administration experiments, overexpression of miR-132 in dentate gyrus increased morphine-seeking behaviors (32), while in a different study, overexpression of miR-9 in the NAc increased oxycodone intake and reduced inter-infusion interval (31). Overall, these results indicate that miRs are important therapeutic targets in SUD.

### Long noncoding RNAs

Long noncoding RNAs (lncRNAs) are a diverse class of RNA molecules that are greater than 200 nucleotides in length and are generally classified based on their genomic location or function (e.g., intronic, intergenic, antisense, and enhancer) (97). Many lncRNAs are expressed in a cell-type and tissue-specific manner and play important regulatory roles in cells by acting as decoy, guide, scaffold, and/or signaling molecules (97, 98). For example, lncRNAs have been shown to mediate gene-specific epigenetic modifications by recruiting chromatin-modifying complexes to their targets (99, 100). At the post-transcriptional level, lncRNAs also fine-tune mRNA splicing, stability, and translation (97). In the mammalian nervous systems, many lncRNAs are highly enriched within the brain and play essential roles in the complex spatio-temporal gene expression mechanisms during brain development and neuroplasticity (98). Consequently, altered lncRNA expression is inherent to several brain diseases, including SUD (10).

One of the first attempts to examine a role for lncRNAs in SUD was made by analyzing lncRNA expression in the NAc of post-mortem heroin- and cocaine-using subjects (101). Relative to drug-free controls, an upregulation of *MIAT*, *NEAT1*, *MALAT1*, and *MEG3* lncRNAs was observed in the NAc of heroin-using subjects, and *MIAT*, *MALAT1*, *MEG3*, and *EMX2OS* upregulation was observed in the NAc of cocaine-using subjects. These well-studied lncRNAs contribute to various cellular processes, including GABA neuron neurogenesis, synapse formation, and cAMP signaling (102–104). In rodent studies, transcriptional profiling of lncRNAs in the NAc of methamphetamine-treated mice revealed thousands of lncRNAs that were altered, mostly downregulated by methamphetamine (105). Further bioinformatic analysis revealed that several of these lncRNAs act as potential cis- or trans-regulators of protein-coding genes involved in reward and addiction pathways. In other experiments, lncRNAs, including *H19*, *Mirg*, *BC1*, *Lrap*, and *Gas5* have also been linked to SUDs (27, 106–110). Although most SUD-related lncRNA experiments have been limited to correlational data, Xu et al. recently revealed a functional role for the lncRNA *Gas5* in SUD models (111). In these studies, cocaine exposure (intraperitoneal injections and self-administration) reduced *Gas5* expression in the NAc, and in behavioral experiments, viral-mediated overexpression of *Gas5* in the NAc attenuated cocaine CPP and self-administration. At the transcriptomic level, *Gas5*-regulated gene expression patterns overlapped significantly with genes altered by cocaine exposure, an indication that *Gas5* regulates cocaine-induced transcriptional responses.

Natural antisense transcripts (NATs) are a class of lncRNAs that have also been implicated in SUD (112). NATs are transcribed from the opposite (antisense) strand of a coding gene and partially or completely overlap with the body, promoter, or enhancer region of the coding gene. Many genes involved in drug-induced neuroplasticity contain NATs (113), and the expression of multiple NATs such as *Bdnf-AS*, *Homer1-AS*, *Traf3ip2-AS1*, and *Prkcq-AS1* is altered by drugs of abuse (35, 113, 114). Therefore, NAT inhibition could be a particularly useful approach to increase the expression of SUD-related protein-coding genes. As a proof of concept, researchers have found that knockdown of *Bdnf-AS* in the infralimbic cortex *via* antisense oligonucleotides attenuated nicotine self-administration (115), and in other experiments, siRNA-mediated silencing of *Bdnf-AS* attenuated ketamine-induced neurotoxicity (116). Thus, with their high target specificity and their emerging roles in drug-seeking behaviors, lncRNAs are promising therapeutic targets for SUD.

### Circular RNAs

Circular RNAs (circRNAs) are single-stranded noncoding RNA molecules produced from pre-mRNAs by a non-canonical splicing process called back-splicing, resulting in covalently closed RNA loops. Approximately 20% of mammalian genes express circRNAs, and while these ncRNAs are present in various organs, their enriched expression in the brain makes them an appealing target for the treatment of neuropsychiatric disorders (117, 118). circRNAs play important roles as transcriptional, post-transcriptional, and/or translational regulators through various mechanisms, most notably as a sponge for miRs (119). Compared to linear RNAs, circRNAs are highly stable (120), and thus may also mediate long-term effects in several disease states.

In several recent papers, a role for circRNAs in SUD has been explored. For example, RNA-sequencing analysis of post-mortem human NAc samples identified several circRNA–miR interactions that were associated with alcohol dependence (121), and in rodent studies, prenatal alcohol exposure was shown to alter the expression of brain circRNAs in a sex-specific manner (122). circRNAs are also dysregulated by opioids (24, 33, 123). In particular, CircTmeff-1, a sponge of miR-541–5p and miR-6934–3p, was observed to be functionally important for morphine CPP (24) and more recently for the reconsolidation of cocaine CPP (124). In other psychostimulant studies, 90 mouse striatal circRNAs were differentially expressed following cocaine self-administration (125), and 41 differentially expressed circRNAs were discovered in the dorsolateral prefrontal cortex of post-mortem human subjects with cocaine use disorder (126). Finally, in methamphetamine-induced neurotoxicity models, numerous circRNAs were significantly altered following methamphetamine treatment (127), and knockdown of circHomer1 alleviated methamphetamine-induced toxicity (128). Together, these initial experiments indicate an important and emerging role for circRNAs in drug-induced neuroadaptations.

## Categories of ncRNA-targeting drugs

**FIGURE 1:**
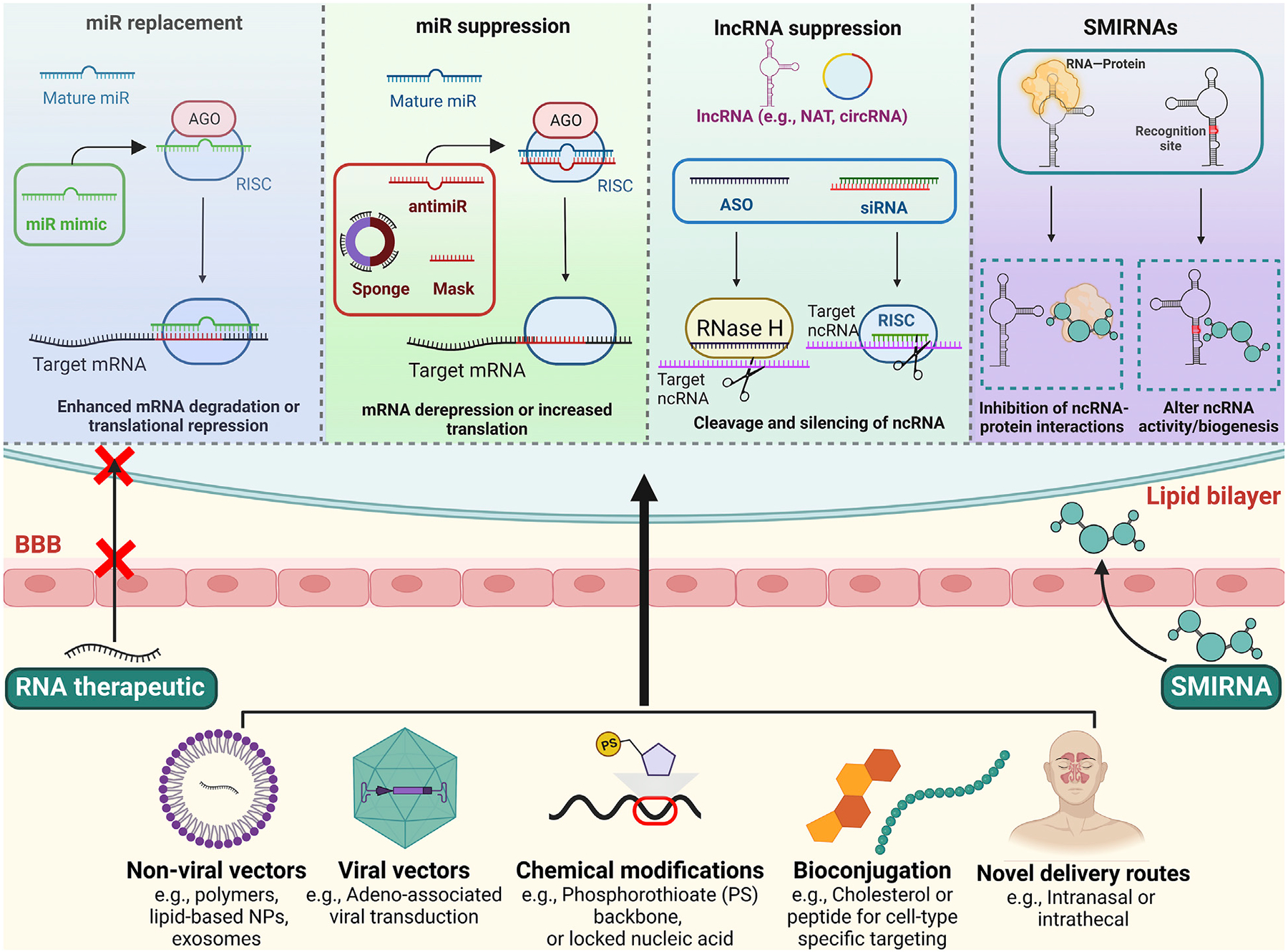
Schematic overview of RNA-targeting therapeutics and strategies to improve CNS delivery. Top: Multiple approaches exist for targeting ncRNAs. For miR replacement, miR mimics are used to imitate endogenous miRs activity, whereas antimiRs, miR masks and sponges inhibit endogenous miR activity. lncRNAs can be targeted with ASOs and siRNAs, leading to their degradation and silencing. SMIRNAs are small molecules that directly bind to ncRNAs or interfere with ncRNA-protein interactions. Bottom: Nanoparticles, viral vectors, chemical modifications, and/or bioconjugations can facilitate stability, cellular uptake, and brain delivery of the ncRNA therapeutics. Novel delivery routes, such as intranasal and intrathecal administration, may also promote CNS delivery and limit systemic toxicities. Some drug-like SMIRNAs are able to cross the blood-brain barrier without a delivery system *via* passive diffusion. Figure created using BioRender.

Due to significant improvements in safety, selectivity and delivery, RNA-based pharmaceuticals have received considerable attention and 14 RNA-based drugs have received FDA or EMA approval since 2015. See reference (129) for a comprehensive review of current FDA-approved RNA therapeutics. In addition to using nucleic acids to target RNAs, researchers have also developed small molecules that target RNA transcripts, termed small molecules interacting with RNA (SMIRNAs) (130). While the initial strategies to target RNAs focused on coding genes, many preclinical and clinical studies are now using similar approaches to target ncRNAs (Figure 1). In this section, we will briefly review the major categories of ncRNA-targeting drugs and highlight potential therapeutic opportunities for each platform in the context of SUD.

### Antisense oligonucleotides

Antisense oligonucleotides (ASOs) are small, synthetic single-stranded nucleic acid molecules that hybridize with the target RNA to alter splicing or translation *via* steric block or RNA degradation (7). The smaller size and stringent binding specificity give ASOs a therapeutic advantage in CNS-related diseases compared to other nucleic acid drugs (Table 2). Indeed, several ASOs that are in clinical trials are being used to treat CNS-related diseases (131). Also, unlike siRNAs, ASOs are able to increase target protein expression by promoting alternative splicing, a strategy that is used clinically for Duchenne muscular dystrophy and spinal muscular atrophy (132).

Unmodified or naked ASOs display significant immunogenicity, low stability, and are rapidly cleared from circulation (133). Thus, chemical modifications are necessary to improve pharmacokinetics and pharmacodynamics of ASO pharmaceuticals (for a comprehensive review see (134)). Common ASO modifications include substitution of a phosphorothioate (PS) backbone and sugar moiety modifications at 2′ position (e.g., 2′-O-methyl, locked nucleic acid, LNA) (134). Though each type of chemically modified ASOs has advantages and disadvantages, in general, these modifications increase safety, stability, and affinity while reducing the need for delivery systems. However, because most ASOs and other nucleic acids are unable to cross the blood-brain barrier, intrathecal or intranasal administration is typically required to target the CNS (135). Currently, there is at least one ncRNA-targeting ASO undergoing clinical testing for Angelman syndrome (NCT05127226) after successful *in vitro* and *in vivo* investigations (136). A few SUD-associated lncRNAs (e.g., *MALAT1*, *MIAT*, and *BDNF-AS*) have been successfully targeted using ASOs in other preclinical disease models (137–141), but additional work is needed to determine if these or other ncRNA-targeting ASO formulations are effective in SUD models.

### siRNAs

SiRNAs are short double-stranded RNAs that attach to RISC, unfold, and form Watson-Crick base pairing with the target RNA, leading to argonaute-induced degradation of the transcript (129). Like ASOs, chemical modifications to siRNAs have improved their safety and efficacy (142–144) and currently 5 siRNA-based drugs have received FDA or EMA approval (Patisiran, Givosiran, Lumasiran, Inclisiran, Vutrisiran). However, in contrast to some ASOs, siRNA platforms depend on the intracellular machinery for their effects, which may restrict the type and number of chemical modifications to the siRNA. Also, in some instances, siRNAs are not as effective at targeting nuclear RNAs compared to ASOs (145), and because of their larger size and negative charge, unmodified siRNAs require the use of a delivery agent to enter the cell (Table 2). To combat some of these limitations, researchers have developed siRNA prodrugs (siRibonucleic neutrals, siRNNs) that disguise the siRNAs’ negative charge by replacing phosphodiesters with charge-neutral phosphotriesters (146). These siRNA prodrugs are able to cross the lipid bilayer, and once in the cell, the phosphotriester group is cleaved off by thioesterases, allowing for the knockdown to occur.

While most FDA-approved siRNA drugs target the liver, there has been a growing interest in using novel siRNA formulations to treat CNS-related disorders. For example, Regeneron Pharmaceuticals and Alnylam Pharmaceuticals recently announced a billion-dollar collaboration to develop siRNA-based drugs for CNS indications (147). Further supporting the usefulness of siRNA-based drugs for CNS uses, recent preclinical experiments identified novel, chemically modified siRNAs that exhibited safe, potent, and long-lasting gene silencing in the brain of rodents and nonhuman primates following intrathecal administration (148). Using systemic or direct brain injections, siRNA-targeting of ncRNAs has been achieved in animal models of SUD (124, 149, 150), Parkinson’s disease (151, 152), Alzheimer’s disease (153–155), epilepsy (156, 157) and stroke (158, 159). Thus, with recent FDA approvals, multiple ongoing, late-stage clinical tests, and promising preclinical data, siRNA-based therapeutics appear to have a promising future, but more testing of siRNA formulations for CNS indications is needed.

### miR replacement/suppression

MiR targeting has been achieved using RNA interference approaches. For example, miR mimics are modified double-stranded RNA molecules that imitate endogenous miR activity and bind to the 3′UTR region of the target mRNAs (37). This approach leads to a downregulation of the target mRNAs *via* translational inhibition. On the other hand, antimiRs, miR sponges, and miR masking techniques are used to reduce miR activity. Structurally similar to ASOs, miR inhibitors or antimiRs prevent an endogenous miRs interaction with its target genes. These single-stranded molecules are usually modified using locked nucleic acid, peptide nucleic acids, or cholesterol (i.e., antagomiR) to improve stability, cellular uptake, and *in vivo* delivery (134, 160). To inhibit a family of miRs, miR sponges, synthetic transcripts that contain various complementary sequences that recognize the seed sequences of multiple miRs, have also been employed in preclinical studies (161–163). Finally, in a technique called miR masking, ASOs bind to 3′UTR sites on a specific mRNA and prevent its interaction with a complementary miR (21) (Figure 1).

In preclinical studies, researchers have demonstrated the effectiveness of antimiRs in animal models of alcohol (19, 164–166), cocaine (25, 124), and opioid (167) use disorders *via* intrathecal or direct brain injections. In other disease models, SUD-relevant miRs (miR-34, miR-145, miR-212) have been targeted with miR mimics (168–170). Although miR-based therapeutics have yet to be tested clinically in SUD patients, several miR mimic and antimiR formulations are being tested in animals or clinical trials for other diseases (171–175). To move the field of miR-targeted SUD therapeutics forward, researchers are encouraged to identify miRs that drive relapse and craving (rather than acquisition of drug-seeking behaviors) and test clinically relevant miR-targeted formulations in sophisticated SUD models.

### Small molecules interacting with RNAs

Emerging research indicates that the three-dimensional structure of RNA, which creates well-defined recognition sites and motifs, can be selectively targeted with small molecules (176). Other than directly binding to specific RNAs (including ncRNAs), SMIRNAs are also able to indirectly affect the RNA functions by interfering with RNA biogenesis or RNA-protein interactions (177–179) (Figure 1). Unlike nucleic acid-based treatments, many SMIRNAs have low molecular weights (usually <1 KDa) and may be administered orally (180), important factors for translational applications (Table 2). However, the likelihood of discovering a small molecule with favorable drug-like characteristics depends on the selected RNA target (181). In other words, the RNA must contain a unique recognition site with considerable structural complexity, differentiating it from other RNAs to avoid non-specific binding and side effects. Also, the abundance of the RNA may influence the efficacy of SMIRNAs (182), a potential issue when targeting very low expressing lncRNAs.

Despite the aforementioned challenges, several SMIRNAs have been identified and validated in preclinical studies (183–189), and in 2020 Risdiplam (an orally available, non-antibiotic SMIRNA) received FDA approval for the treatment of spinal muscular atrophy (190). ncRNAs have also been successfully targeted with SMIRNAs. For example, two studies have identified SMIRNAs for MALAT1 (191, 192), a lncRNA that is altered in the brain of heroin and cocaine users and in rats treated with morphine (101, 193). In other studies, a first-in-class, clinical-stage quinolone compound, ABX464, was found to increase the expression of miR-124, a target that has been well-studied in SUD models (194). This molecule has passed phase I dose safety trial and phase IIa clinical studies, and although ABX464 has been mainly studied in HIV and Ulcerative Colitis, it could also be used to upregulate miR-124 expression in the brain to reduce drug-induced neurobehavioral adaptations (194). NPC86 is another SMIRNA that stabilizes the lncRNA *Gas5* (195), a lncRNA that has been associated to cocaine-seeking behaviors (111). Finally, the *let-7* family, miRs with a known link to SUD, are suppressed by RNA-binding proteins called LIN28. Recently, Wang et al. successfully identified six small molecule disruptors of LIN28 and subsequently *let-7* suppression (179). Together, these studies indicate that targeting ncRNAs with SMIRNAs is a feasible approach and may have potential utility in SUD.

## Delivery systems for ncRNA therapeutics

Despite several advances, treating CNS diseases with nucleic acids-based platforms remains a major challenge due to the blood-brain barrier. Comprised of tight junctions between brain capillary endothelial cells, the blood-brain barrier prevents large molecule therapeutics from entering the brain parenchyma. To circumvent this issue, researchers have developed several RNA delivery systems that are capable of entering the brain *via* intravenous, intrathecal, or intranasal routes of administration (131, 196–200). Viral vectors and nanoparticle carrier systems are some of the most promising strategies for delivering ncRNA therapeutics to the brain and are discussed below.

### Viral vectors

In preclinical studies, viral vectors are widely used to transfer nucleic acids to brain cells with high efficiency (201). The most commonly used viral vectors for delivering nucleic acids are adenovirus, adeno-associated virus (AAV), and lentivirus vectors (202–204). In neuroscience research, AAVs are especially popular as different serotypes allow for transduction of distinct brain cells (205) and projection-specific pathways (206). Another advantage of viral vectors is the ability to target disease-related brain cells, using cell type-specific promoters (207, 208). However, the vast majority of SUD-related studies that have used viral vectors to manipulate ncRNAs have done so by direct brain injections, an approach that may have limited clinical utility. More recently, researchers have developed viral vectors that are capable of targeting the brain *via* more feasible routes of administration. For example, intrathecal injection of an AAV that expresses an artificial miR resulted in robust gene silencing with no observed side effects in nonhuman primates (209). Using the same approach, a case study in ALS patients also generated promising results (210). In animal models of Huntington’s disease, intravenous injection of a novel AAV encoding an artificial miR that targets the huntingtin (HTT) gene yielded extensive knockdown of HTT across multiple brain regions with the highest transduction observed in the striatum (199). Several other studies have also explored viral-mediated CNS delivery of ncRNAs *via* intrathecal or intravenous routes of administration (211–215) and multiple clinical trials using AAVs in Parkinson’s disease, Alzheimer’s disease, Batten disease, and Canavan disease patients have been conducted or in progress (216). In summary, nonpathogenic viral vectors offer a powerful option for ncRNA-targeted brain delivery and should be further pursued in SUD patients.

### Nanoparticles

Nanoparticle-mediated delivery of ncRNA therapeutics is a promising approach for the treatment of SUD (217). Nanoparticles have several appealing properties including, tunable release rate, biocompatibility, limited toxicity, brain penetrating capabilities, and adjustable surface modifications for cell type-specific delivery (218). Many different classes of nanocarriers have been successfully tested in CNS disease models, including polymeric, inorganic, exosome, and lipid-based nanoparticles (219–229), and as an indication of their safety and efficacy across multiple disease states, several nanoparticle formulations have received FDA approval, including the recent approvals of the Pfizer-BioNTech and Moderna COVID-19 vaccines (both use lipid nanoparticles for mRNA delivery) and the siRNA drug Patisiran (230).

Although nanoparticle-mediated brain delivery *via* systemic administration remains an ongoing challenge, miR mimic and antimiR encapsulated nanoparticles have successfully targeted the brain in multiple CNS disease models following systemic administration (231–233). For example, intranasal delivery of extracellular vesicles loaded with miR-124 to cocaine-treated mice resulted in successful brain uptake and abrogation of inflammatory markers (234). A more recent strategy for the delivery of nucleic acids to the brain is to add surface modifications to the nanoparticles that facilitate transport across the blood-brain barrier. For example, using sugar-coated polymeric nanoparticles that bind a major glucose transporter in the brain called GLUT-1, researchers successfully targeted coding and noncoding transcripts in the brain following intravenous administration (225, 235). In other studies, exosomes with a transferrin binding ligand attached to the surface effectively delivered antimiRs into the rat brain after an intravenous injection. Systemic delivery of nucleic acid payloads to the brain has also been accomplished using rabies virus glycoprotein (RVG) exosomes and liposomes (236–238), transferrin-targeted cyclodextrins (239), angiopep-2-targeted lipid- and polymer-based nanoparticles (240, 241), and calcium phosphate lipid nanoparticles (242). Thus, as the number of nanoformulations capable of delivering nucleic acids to the brain continues to improve, ncRNA nanocarrier systems warrant further research in SUD models.

## Ongoing challenges and outlook

The lipid bilayer is a billion-year-old barrier that prevents large, charged molecules like RNAs from entering the cell. In addition to this barrier, there are other formidable obstacles that protect cells from RNAs including, RNases, the innate immune system, and for neurons, the blood-brain barrier (243). Despite these natural defenses, decades of basic science and clinical research have recently led to multiple FDA-approved nucleic acid-based therapeutics for various indications (244). However, it is clear that we are still in the early days of ncRNA therapeutic development, particularly for SUD, and several issues need to be addressed to move the field forward. First, most preclinical and all clinical experiments exploring ncRNAs in SUD are correlational studies. Additional functional studies that target conserved ncRNAs in sophisticated SUD models will be essential to identify the ncRNA targets with the highest translational potential. Also, as low-quality sequence data have incorrectly annotated some ncRNAs (245, 246), SUD-associated ncRNAs should be thoroughly characterized and validated as true ncRNAs before being pursued therapeutically. To facilitate therapeutic developoment, multiple bioinformatic tools have been created to predict ncRNA targets and assist with characterization and safety (245, 247). Second, rather than studying the ncRNAs involved in the acquisition of drug-seeking, researchers should focus on ncRNA mechanisms that drive drug craving, relapse, and withdrawal, as such targets are likely more relevant to promote abstinence and recovery in humans. Also, as different cells and circuits may exert contrasting effects in the context of SUD, additional cell-type specific studies are needed to identify the most promising ncRNA targets. Third, instead of injecting RNA-based therapeutics directly into the brain in preclinical models, researchers are encouraged to test clinically relevant routes of administration for ncRNA treatments. For example, multiple studies have demonstrated the promise of intranasal administration as a way to bypass the blood-brain barrier (196, 197, 231, 248–253). Intrathecal injections of modified ASOs and siRNAs and nanoparticle-containing nucleic acids have also achieved high brain uptake in preclinical and clinical studies (131, 200, 254) and should also be employed in SUD experiments. Finally, using nucleic acids, nanoparticles, and/or AAVs that contain ligands or surface modifications to promote brain and/or cell type-specific delivery is an approach to enhance CNS uptake and avoid potential side effects (7, 217, 247, 255–257). N-acetylgalactosamine (GalNac), a biomolecule conjugate that promotes liver-specific uptake of RNA-targeted therapeutics, is a prime example of how such modifications can facilitate tissue-specific uptake. Additional research is needed to determine whether similar opportunities exist to enhance CNS-specific delivery.

An additional strategy to move the field forward is to repurpose or test clinical-stage nucleic acid-based therapeutics that may also have relevance to SUD. For example, several companies have developed miR mimics or antimiR that target miRs linked to SUD (28, 53, 64, 73, 74, 82, 83, 258–260). Also, SMIRNA databases (e.g., R-BIND, infoRNA) (261, 262) could be used to identify compounds that target SUD-relevant ncRNAs, an appealing translational approach as small molecules typically have a better physicochemical profile compared to nucleic acids. These databases also consist of clinically tested small molecules, providing drug repurposing opportunities for rapid translational applications. Additionally, the abused substance itself may create opportunities for nucleic acid-based treatments. For example, the disrupted blood-brain barrier caused by chronic methamphetamine use (263) may allow for RNA-based drug delivery *via* less invasive routes of administration, a hypothesis that merits further exploration.

Although many promising opportunities are listed above, multiple clinical trials using RNA-based treatments have been withdrawn due to severe side effects or limited efficacy (18, 247, 264). These failures may serve as lessons learned for future SUD therapeutics. For instance, in preclinical studies, MRX34, a liposome-delivered miR-34a mimic for treatment of solid tumors, showed favorable efficacy and safety profile (265, 266). However, when injected systemically in humans, MRX34 induced severe immune-related side effects and death in some patients causing the clinical trial to be terminated (264). MRX34 was designed to target the low-pH environment in tumors, but preclinical studies indicated that it also accumulates in the bone marrow and other organs, potentially impacting immune cell activity (267). This incident highlights the need for a thorough risk assessment of all organ systems following systemic administration of RNA therapeutics. In another example, oblimersen, a phosphothiorate-modified ASO targeting *BCL2* mRNA, showed promise in preclinical experiments but lacked efficacy in multiple clinical trials (268, 269). Further analyses revealed that several off-target effects of oblimersen were related to the phosphothiorate modification, as these off-target effects were not observed with the same ASO that lacked this modification (270–272). On a related note, the RNA payload may also alter the efficacy of the delivery vehicle. For example, nanoparticle tropism has been shown to change based on the type of cargo (273). Thus, going forward, each RNA modification along with the delivery vehicle should be carefully assessed for efficacy and safety before moving to human subjects.

Dosing is another major issue that needs to be addressed in ncRNA-targeting therapeutics, as many ncRNA studies have used supraphysiological concentrations that may lead to unpredictable side effects (247, 274). For example, high doses of miR mimics can cause off-target effects by saturating RISC, potentially blocking the activity of unrelated miRs and triggering a cascade of side effects. As a prerequisite for clinical studies, future experiments should establish dose-dependent on- and off-target effects of the ncRNA therapeutic in both control and pathological conditions. To address dose-dependent toxicities, metronomic ncRNA therapy is an approach used in cancer in which frequent low doses of the ncRNA therapy are administered (usually in combination with conventional treatments) to avoid excessive toxicity or immunogenicity (275). Similar strategies could also be investigated for efficacy and safety in SUD studies. Finally, the exorbitant price of RNA-based therapeutics is a continuing issue that needs to be addressed, particularly for SUD patients that may lack sufficient means to purchase these costly drugs. Ongoing efforts to address these concerns will open the door for ncRNA SUD therapeutics.

## Figures and Tables

**TABLE 1:** Examples of ncRNA modulation in animal models of SUD.

ncRNA	Drug	Model	Region	Modality	Change	Reference
Let-7d	Alcohol	TBC	NAc	LV-let7d	↓ Intake	(18)
miR-30a-5p	Alcohol	TBC	mPFC	AdVs miR-30a-5p	↑ Intake	(19)
				LNA antimiR-30a-5p	↓ Intake	
miR-124a	Alcohol	TBC and CPP	DLS	LV-si124a	↓ Intake and CPP	(20)
				LV-miR124a	↑ Intake and CPP	
miR-137	Alcohol	EPM	AMG	LNA-antimiR-137	↓ Anxiety and consumption behaviors	(21)
miR-382	Alcohol	TBC	NAc	AdV-miR-382	↓ Intake	(22)
Let-7d	Cocaine	CPP	NAc	LV-silet7d	↑ CPP	(23)
				LV-miR-let7d	↓ CPP	
miR-124a	Cocaine	CPP	NAc	LV-si124	↑ CPP	(23)
				LV-miR-124	↓ CPP	
miR-181a	Cocaine	CPP	NAc	LV-si181a	↓ CPP	(23)
				LV-miR-181a	↑ CPP	
miR-206	Cocaine	CPP	NAc	AntagomiR-206	↑ CPP	(24)
miR-212	Cocaine	SA	DS	LV-miR212	↓ Intake	(25)
				LNA-antimiR-212	↑ Intake	
miR-495	Cocaine	SA	NAc	LV-miR495	↓ Seeking behavior	(26)
Gas5 lncRNA	Cocaine	CPP	NAc	AAV-Gas5 or HSV-Gas5	↓ Intake and CPP	(27)
circTmeff-1	Cocaine	CPP	NAc core	AAV-siR-circTmeff-1	↓ CPP	(24)
miR-29c	METH	OFT	NAc	AAV-miR-29c	↑ Locomotor activity	(28)
				AAV-antimiR-29c	↓ Locomotor activity	
miR-31–3p	METH	CPP	dHIP	AAV-miR-31–3p	↑ CPP	(29)
				AAV-antimiR-31–3p	↓ CPP	
miR-128	METH	OFT	NAc	AAV-miR128	↑Locomotor activity	(30)
				AAV-antimiR128	↓Locomotor activity	
miR-9	Oxycodone	SA	NAc	AAV-miR-9	↑ Intake	(31)
miR-132	Morphine	SA	DG	LV-miR-132	↑ Seeking behavior	(32)
circTmeff-1	Morphine	CPP	NAc core and shell	AAV-siR-circTmeff-1	↓ CPP	(33)
				AAV- circTmeff-1	No effect on CPP	
miR-221	Nicotine	EEM	mPFC	LV-miR-221	↑ Locomotor activity	(34)
BDNF-AS	Nicotine	SA	ILC	Anti BDNF-IV-AS ASO	↓ Drug-induced Reinstatement	(35)

AdV, adenoviral; AMG, amygdala; DG, dentate gyrus; DS, dorsal striatum; DLS, dorsolateral striatum; dHIP, dorsal hippocampus; EEM, enriched environment model; EPM, elevated plus maze; HSV, herpes simplex virus; ILC, infralimbic cortex; LV, Lentiviral; LNA, locked nucleic acid; METH, methamphetamine; mPFC, medial prefrontal cortex; OFT, open field test; SA, self-administering; siR, silencer; TBC, two-bottle choice.

**TABLE 2:** Characteristics of RNA-targeting drugs for CNS indications.

Characteristics	ASOs	siRNAs	SMIRNAs
**Target**	Nucleic acid	Nucleic acid	Nucleic acid or protein
**Effect on ncRNA**	Increase/decrease activity	Decrease activity	Increase/decrease activity
**Duration of effect**	Days to weeks	Days to weeks	Hours
**Specificity and Strength**	Specific and potent	Specific and potent	Specificity and potency vary
**Lead optimization**	Rapid	Rapid	Slow
**Drug-likeness**	Chemical modifications needed	Chemical modifications and/or delivery systems needed	Drug-like
**Route of Administration**	Usually intrathecal	Usually intrathecal	Usually oral
**Manufacturing cost**	High cost but lower than siRNAs	High cost	Lower cost
